# β-Cell Insulin Resistance Plays a Causal Role in Fat-Induced β-Cell Dysfunction In Vitro and In Vivo

**DOI:** 10.1210/endocr/bqae044

**Published:** 2024-04-05

**Authors:** Aleksandar Ivovic, Justin Hou Ming Yung, Andrei I Oprescu, Filip Vlavcheski, Yusaku Mori, S M Niazur Rahman, Wenyue Ye, Judith A Eversley, Michael B Wheeler, Minna Woo, Evangelia Tsiani, Adria Giacca

**Affiliations:** Department of Physiology, Faculty of Medicine, University of Toronto, Toronto, ON M5S 1A8, Canada; Department of Physiology, Faculty of Medicine, University of Toronto, Toronto, ON M5S 1A8, Canada; Institute of Medical Science, Faculty of Medicine, University of Toronto, Toronto, ON M5S 1A8, Canada; Department of Health Sciences, Brock University, St. Catharines, ON L2S 3A1, Canada; Department of Physiology, Faculty of Medicine, University of Toronto, Toronto, ON M5S 1A8, Canada; Division of Diabetes, Metabolism, and Endocrinology, Anti-Glycation Research Section, Department of Medicine, Showa University School of Medicine, Shinagawa, Tokyo 142-8555, Japan; Department of Physiology, Faculty of Medicine, University of Toronto, Toronto, ON M5S 1A8, Canada; Department of Physiology, Faculty of Medicine, University of Toronto, Toronto, ON M5S 1A8, Canada; Department of Physiology, Faculty of Medicine, University of Toronto, Toronto, ON M5S 1A8, Canada; Department of Physiology, Faculty of Medicine, University of Toronto, Toronto, ON M5S 1A8, Canada; Institute of Medical Science, Faculty of Medicine, University of Toronto, Toronto, ON M5S 1A8, Canada; Toronto General Hospital Research Institute, University Health Network, University of Toronto, Toronto, ON M5G 2C4, Canada; Division of Endocrinology, Department of Medicine, University Health Network, University of Toronto, Toronto, ON M5G 2C4, Canada; Department of Medicine, Faculty of Medicine, University of Toronto, Toronto, ON M5S 1A8, Canada; Banting and Best Diabetes Centre, University of Toronto, Toronto, ON M5G 2C4, Canada; Department of Health Sciences, Brock University, St. Catharines, ON L2S 3A1, Canada; Department of Physiology, Faculty of Medicine, University of Toronto, Toronto, ON M5S 1A8, Canada; Institute of Medical Science, Faculty of Medicine, University of Toronto, Toronto, ON M5S 1A8, Canada; Department of Medicine, Faculty of Medicine, University of Toronto, Toronto, ON M5S 1A8, Canada; Banting and Best Diabetes Centre, University of Toronto, Toronto, ON M5G 2C4, Canada

**Keywords:** beta-cell dysfunction, beta-cell insulin signaling, beta-cell insulin resistance, lipotoxicity, oleate, olive oil

## Abstract

In the classical insulin target tissues of liver, muscle, and adipose tissue, chronically elevated levels of free fatty acids (FFA) impair insulin signaling. Insulin signaling molecules are also present in β-cells where they play a role in β-cell function. Therefore, inhibition of the insulin/insulin-like growth factor 1 pathway may be involved in fat-induced β-cell dysfunction. To address the role of β-cell insulin resistance in FFA-induced β-cell dysfunction we co-infused bisperoxovanadate (BPV) with oleate or olive oil for 48 hours in rats. BPV, a tyrosine phosphatase inhibitor, acts as an insulin mimetic and is devoid of any antioxidant effect that could prevent β-cell dysfunction, unlike most insulin sensitizers. Following fat infusion, rats either underwent hyperglycemic clamps for assessment of β-cell function in vivo or islets were isolated for ex vivo assessment of glucose-stimulated insulin secretion (GSIS). We also incubated islets with oleate or palmitate and BPV for in vitro assessment of GSIS and Akt (protein kinase B) phosphorylation. Next, mice with β-cell specific deletion of PTEN (phosphatase and tensin homolog; negative regulator of insulin signaling) and littermate controls were infused with oleate for 48 hours, followed by hyperglycemic clamps or ex vivo evaluation of GSIS. In rat experiments, BPV protected against fat-induced impairment of β-cell function in vivo, ex vivo, and in vitro. In mice, β-cell specific deletion of PTEN protected against oleate-induced β-cell dysfunction in vivo and ex vivo. These data support the hypothesis that β-cell insulin resistance plays a causal role in FFA-induced β-cell dysfunction.

Obesity and type 2 diabetes are associated with a chronic elevation of free fatty acids (FFA), which are known to impair β-cell function. The mechanisms involved are not completely understood, but major contributors include oxidative stress, endoplasmic reticulum stress, and inflammation (1-3). In insulin target tissues such as muscle, liver, and adipose tissue, these pathways are the mechanisms whereby FFA impair insulin signaling (4-7), due to activation of inflammatory kinases (8, 9). Similar mechanisms are involved in FFA-induced β-cell dysfunction, and it is possible that they lead to insulin resistance in β-cells.

When the β-cell insulin signaling pathway is activated, which involves insulin receptor substrate (IRS) tyrosine phosphorylation followed by stimulation of the phosphoinositide 3-kinase/ protein kinase B (PI3K-Akt) pathway, Akt can mediate its effects such as β-cell growth and proinsulin biosynthesis (10, 11). Activated Akt inhibits forkhead box protein O1 (FOXO1), which leads to increased pancreatic and duodenal homeobox 1 (PDX-1)-mediated transcription of the insulin gene (11-13).

The effect of insulin on insulin secretion specifically is less clear. Early studies found that incubation of β-cells with insulin stimulated insulin secretion via an IRS-1-dependent mechanism that leads to increased cytosolic calcium (14-16). In contrast, other studies have shown that PI3K inhibits insulin secretion due to opening of K_ATP_ channels by phosphatidylinositol-34,5-trisphosphate (PIP3) (17-19). In vivo, the prevailing effect of insulin appears to be stimulatory, as β-cell specific insulin receptor knockout (βIRKO) mice show a defect in first-phase insulin secretion (20).

In the context of fat exposure, in vitro studies have shown that incubation of INS-1 cells with FFA impairs the insulin-like growth factor (IGF)-1 induced activation of Akt (21-23), decreases Akt-mediated mitogenesis (22), and induces β-cell apoptosis (21). Palmitate-induced JNK activation was also found to decrease IRS-1 and IRS-2 tyrosine phosphorylation and in turn to impair insulin gene transcription (24). Furthermore, adenoviral-mediated overactivation of Akt prevented oleate-induced apoptosis (21). In addition, some of our group have shown that β-cell specific deletion of the negative regulator of insulin signaling PTEN (phosphatase and tensin homolog), which dephosphorylates PIP3 to phosphatidylinositol 4,5-bisphosphate (PIP2), protects against obesity-induced diabetes in both dietary (high fat diet) and genetic (*db*/*db* mice) models of type 2 diabetes (25). Although β-cell PTEN knockout mice have increased % β-cell/total pancreatic area (index of β-cell mass), as expected due to the chronic increase in growth-promoting pathways such as Akt and mTOR (25), their protection against obesity-induced diabetes in both of these models is due to improved β-cell function as evaluated by islet perifusion studies (25). With a high fat diet, it is impossible to isolate the effect of obesity or gastrointestinal effects from the effect of fat per se on β-cell function. In this study, we wished to determine whether upregulation of insulin signaling by the insulin mimetic bisperoxovanadate (BPV) as well as by β-cell specific PTEN deletion, would protect against β-cell dysfunction induced specifically by elevation of FFA. Vanadium compounds are nonspecific tyrosine phosphatase inhibitors; thus, they can be used to upregulate insulin signaling. These compounds, unlike other insulin sensitizers such as metformin and thiazolidinediones, increase rather than decrease oxidative stress (26). These other insulin sensitizers are also AMP kinase activators and thus deplete islets of fat (27). Therefore, a positive effect in preventing lipotoxicity by BPV could not be ascribed to reduction of oxidative stress or islet fat.

In order to address our hypothesis that upregulation of insulin signaling would protect against β-cell dysfunction specifically induced by fat, we intravenously infused rats with oleate or olive oil as in our previous study (28) with and without BPV, followed by hyperglycemic clamps for in vivo assessment of β-cell function or assessment of glucose-stimulated insulin secretion (GSIS) ex vivo in islets. Rat islets were also incubated with oleate or palmitate with or without BPV in vitro for assessment of GSIS and Akt phosphorylation. However, as mentioned above, BPV upregulates insulin signaling via nonspecific inhibition of tyrosine phosphatases, and the intravenous infusion of BPV will have effects in other tissues besides β-cells. Thus, we utilized another model, that is, the β-cell PTEN (PIP3 phosphatase) knockout mouse to upregulate insulin signaling in a tissue-specific manner. Although also this model is relatively nonspecific with regard to upregulation of insulin signaling, our group has previously shown that chronic activation of the PI3K/Akt pathway in these mice (25) does not affect tumorigenesis (29), islet architecture, or DNA repair (25). We infused β-cell specific PTEN-knockout mice with oleate, followed by in vivo hyperglycemic clamps or ex vivo determination of GSIS in islets. In rats, we found that BPV protected against oleate- and olive oil–induced β-cell dysfunction in vivo and ex vivo and against oleate- and palmitate-induced β-cell dysfunction in vitro. In addition, BPV prevented the FFA-induced reduction of insulin-stimulated Akt activation in rat islets. In mice, β-cell specific deletion of PTEN prevented fat-induced β-cell dysfunction both in vivo and ex vivo.

## Methods

### Animals

All procedures were in accordance with the Canadian Council of Animal Care Standards and were approved by the Animal Care Committee of the University of Toronto. Female Wistar rats (250-300 g, Charles River, Canada) were used for BPV experiments, as in our previous studies (28). PTEN floxed mice were crossed with RIP2-Cre-positive C57BL/6J mice in order to generate homozygous and heterozygous β-cell specific PTEN knockout mice, homozygous PTEN floxed RIP2-Cre negative controls, and wild-type PTEN RIP2-Cre-positive controls. Male mice were used. Results in the 2 control groups were combined, as there was no difference between them. The PTEN floxed breeders were on a C57BL/6-129J genetic background; however, unlike in the study by Wang et al (25), and similar to the study by Stiles et al (30), their offspring were not deliberately maintained on this mixed background, being backcrossed to C57BL/6J for at least 2 generations. All animals were housed in the University of Toronto's Department of Medicine, exposed to a 12-hour light/dark cycle and fed standard rodent chow containing 24% protein, 58% carbohydrate, and 18% fat by calorie content (Teklad Global 2018, Madison, WI, USA).

### Cannulation Surgeries and Intravenous Infusions

The jugular vein and carotid artery were cannulated in rats as described in our previous studies (28, 31). After 3 days of recovery from surgery, rats were randomized and infused for 48 hours with either (i) saline control (SAL); (ii) oleate (OLE, 1.3 µmol/min) bound to bovine serum albumin (BSA) and prepared as described in our previous studies (28, 32, 33) or olive oil plus heparin (OLO, 50 U/mL heparin; 5.5 µL/min), also as described in our previous studies (28) to elevate plasma FFA by 1.5 to 2-fold (28), which are elevations seen in obesity (34); (iii) oleate plus bisperoxovanadate (BPV, 0.0025 μmol/kg/min, the dose that increased insulin sensitivity in other authors’ studies (35) or OLO plus BPV; or (iv) BPV alone. Saline was used as control, as we have previously shown that there is no difference compared to BSA infusion in the same experimental models (rats and mice) (33, 36). Following the infusions and after overnight fasting, rats either underwent a 2-step hyperglycemic clamp for in vivo assessment of β-cell function or islets were isolated for ex vivo assessment of GSIS as in our previous studies (28, 31, 32).

Control mice (either PTEN floxed controls or wild-type RIP2-Cre-positive controls), β-cell specific heterozygous PTEN-knockout mice, and β-cell specific homozygous PTEN-knockout mice underwent jugular vein cannulation for intravenous oleate infusion and hyperglycemic clamps or ex vivo assessment of GSIS as described in our previous studies (28, 31, 36). At 3 to 5 days after surgery, mice were weighed and infused for 48 hours at a rate of 0.4 µmol/min with oleate or saline followed by either 2 hours in vivo hyperglycemic clamps or islet isolation for ex vivo assessment of GSIS, both carried out after 4 hours fasting.

### Pancreatic Islet Isolation

Islets were isolated from the 48-hour infused rats using the Ficoll/Histopaque method, as described previously (32). Another set of islets were isolated from untreated rats using the same method for in vitro experiments. Pancreatic islets of mice were isolated following 48 hours infusion, as previously described (28, 37).

### Hyperglycemic Clamps, Insulin Sensitivity Index, Disposition Index, and Insulin Clearance Index

Two-step hyperglycemic clamps at 13 mM (upper physiological glucose level) and 22 mM glucose (maximum stimulatory level) in rats and one-step hyperglycemic clamps at 22 mM glucose in mice were performed as described in our previous studies (28, 36). We reached the steady state target glucose level slowly to avoid cardiac arrhythmias potentially induced by a bolus of oleate in the infusion line when suddenly elevating the glucose infusion. The sensitivity index (SI) and disposition index (DI) were calculated as previously described (28, 36). The SI is an index of insulin sensitivity, calculated as glucose infusion rate (GINF)/[Insulin] (38). DI is an established in vivo measure of β-cell function (39, 40), calculated as GINF/[Insulin] * [C-peptide] (28, 36). The insulin clearance index was calculated as [C-peptide]/[Insulin]. Steady state values (last 40 minutes of each step in rats and last 20 minutes in mice) were used for calculation. There are limitations in calculating SI during hyperglycemic clamps, due to the nonlinearity of the relationship of GINF to insulin at high insulin levels (41) and it might be argued that SI and DI should not be calculated from the same experiment; however, it is too invasive to perform both hyperglycemic and hyperinsulinemic clamps in the same rodent.

### Ex Vivo Studies in Islets

Freshly isolated islets of in vivo infused rats and mice were pre-incubated for 1 hour at 37 °C in Krebs-Ringer bicarbonate HEPES (KRBH) buffer supplemented with 2.8 mM glucose. Thereafter, 5 rat islets of approximately the same size were incubated in triplicate in KRBH buffer containing 2.8 mM (non-GSIS), 6.5 mM (basal glucose level in rodents), 13 or 22 mM glucose (as in hyperglycemic clamps) for 2 hours at 37 °C. Similarly, 10 mouse islets of approximately the same size were incubated in duplicate at 6.5 and 22 mM glucose for 2 hours at 37 °C. Insulin concentration in the medium was analyzed using a radioimmunoassay kit specific for rat/mouse insulin (Linco, St. Charles, MO) as previously described (28, 31, 36). Islet insulin content after acid-ethanol extraction (31, 36) was analyzed by a Homogenous Time-Resolved Fluorescence (HTRF) kit (Cisbio, Codolet, France).

### In Vitro Studies in Islets

Islets of untreated rats were cultured for 48 hours in RPMI 1640 without antioxidants, containing 6.5 mM glucose and 0.4 mM palmitate or oleate in 0.5% BSA or 0.5% BSA alone with or without 4 µM BPV, a dose based on previous studies (42). Thereafter, islets were pre-incubated for 1 hour at 37 °C in KRBH supplemented with 2.8 mM glucose. Subsequently, 5 rat islets of approximately the same size were incubated in triplicate in KRBH buffer containing 6.5 mM or 22 mM glucose for 2 hours for the determination of insulin secretion by measuring insulin concentration in the medium with the Linco kit described above.

### Western Blots

We performed Western blotting on cultured rat islets to investigate the activation of various insulin signaling molecules, including Akt, IRS-1, ERK1/2, GSK3, mTOR. Following 48 hours incubation of rat islets with palmitate or oleate in the presence or absence of BPV, islets were treated with 200 nM insulin for 5 minutes to activate insulin signaling. Following insulin exposure, islets were centrifuged and the pellets (∼100 islets per sample) were lysed in an Eppendorf tube containing 100 μL of 1X lysis buffer (CST #9803) supplemented with 1 EDTA-free protease inhibitor cocktail (Roche 11836170001), 1 mM PMSF (Sigma-Aldrich P7626) and 10 mM sodium fluoride and incubated on ice for 45 minutes. The tube was agitated every 5 minutes to promote lysis. The samples were then centrifuged at 13 000 rpm for 10 minutes at 4 °C. The supernatant was collected, and protein concentration was measured using the BCA protein assay (Pierce). Then 15 μg of protein from each islet sample was resolved by SDS-PAGE, transferred to nitrocellulose membranes, and immunoblotted with the primary antibodies listed in Table 1. Secondary HRP-linked antibody (Cell Signaling Technology Cat #7074) and an enhanced chemiluminescence system (BioRad Cat #1705062) were used for detection. The bands obtained from immunoblotting were quantified using ImageJ software (National Institutes of Health, Bethesda, MD, USA). Samples from freshly isolated islets of mice used in hyperglycemic clamps were also used for Western blot analysis, to determine PTEN expression using a primary antibody from Cell Signaling Technology (Cat# 9552) (Table 1).

**Table 1.** List of primary antibodies used in this study

Antibody	Research Resource Identifier (RRID)	Dilution	Host	Company & Catalog number
Phospho-Akt (Ser473)	AB_329825	1:1000	Rabbit	Cell signaling 9271
Total-Akt	AB_329827	1:1000	Rabbit	Cell signaling 9272
Phospho-mTOR (Ser2448)	AB_330970	1:1000	Rabbit	Cell signaling 2971
Total-mTOR	AB_330978	1:1000	Rabbit	Cell signaling 2972
Phospho-p44/42 MAPK (ERK1/2) (Thr202/Tyr204)	AB_331772	1:1000	Rabbit	Cell signaling 4376
Total-p44/42 MAPK (ERK1/2)	AB_330744	1:1000	Rabbit	Cell signaling 9102
Phospho-GSK3β (Ser9)	AB_10013750	1:1000	Rabbit	Cell signaling 5558
Total-GSK3β	AB_490890	1:1000	Rabbit	Cell signaling 9315
Phospho-IRS-1 (Tyr612)	AB_2533768	1:2500	Rabbit	Invitrogen 44-816G
Total-IRS-1	AB_330333	1:500	Rabbit	Cell signaling 2382
β-actin	AB_10950489	1:1000	Rabbit	Cell signaling 8457
HRP-conjugated anti-rabbit antibodies	AB_2099233	1:3000	Goat	Cell Signaling 7074
PTEN	AB_10694066	1:1000	Rabbit	Cell Signaling 9552
β-actin (for PTEN blots) chicken/avian, human, mouse, rat	AB_2714189	1:10 000	Mouse	Santa Cruz Biotechnology Sc-47778
Insulin (for immunohistochemistry)	AB_2716761	1:30 000	Rabbit	Abcam ab181547

### Morphological Studies

We determined % β-cell/total pancreatic area via immunohistochemistry, as described previously (43), in mouse pancreas collected at the end of the hyperglycemic clamp. Given the difficulty of accurately measuring total pancreatic weight in rodents, which is not supposed to be affected by islet-targeted treatments, % β-cell/total pancreatic area was taken as a proxy of β-cell mass, as in our previous studies (25) and as commonly done in the field (β-cell mass is β-cell//total pancreatic area multiplied by total pancreatic weight). Insulin staining was performed using a primary antibody from Abcam as listed in Table 1 after citrate buffer pH 6.0 antigen retrieval in pressure cooker. The ImmPRESS HRP Horse and Rabbit IgG Polymer kit from Vector G was used to visualize the insulin positive area. Areas were quantified with Image J software.

### Plasma Assays

Plasma assays were performed as previously described (31, 36). Briefly, plasma glucose was measured on a Beckman Analyzer II (Beckman, Fullerton, CA, USA) in rats and on a HemoCue Glucose 201 Analyzer (HemoCue Inc., CA, USA) in mice. Plasma FFA were measured with an enzymatic colorimetric kit (Wako Industries, Neuss, Germany). Radioimmunoassays specific for rat/mouse insulin and C-peptide (Linco, St. Charles, MO, USA) were used to determine their plasma concentrations. All assays were performed in duplicate.

### Statistics

Data are means ± standard error of the mean (SEM). One-way nonparametric ANOVA for repeated measurements followed by Tukey's test was used to compare treatments. Calculations were performed using SAS (Cary, NC, USA).

## Results

### Studies in Rats

#### In vivo hyperglycemic clamps

**Figure 1.**
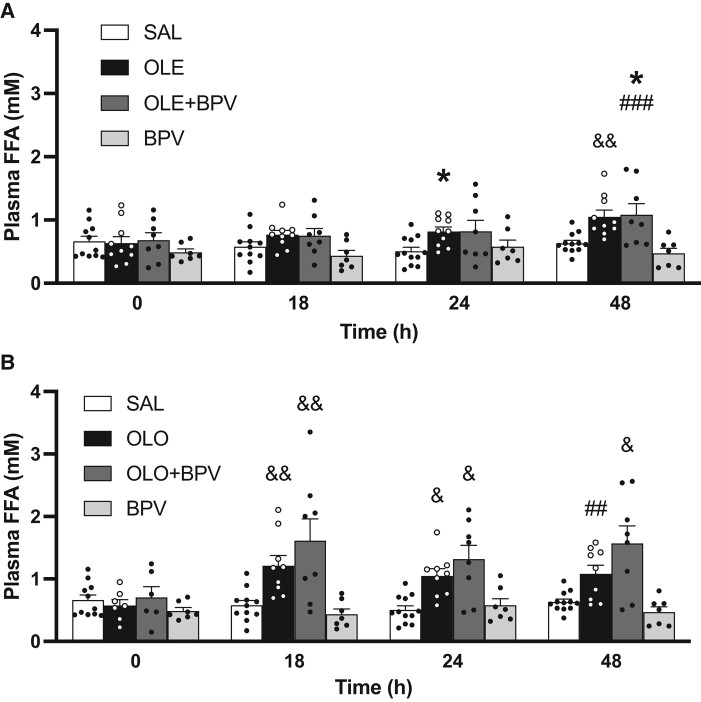
Plasma FFA levels during the 48-hour intravenous infusion period. 12-week-old female Wistar rats were treated with: (i) Saline (SAL, n = 12) (A, B); (ii) Oleate alone (OLE, 1.3 µmol/min, n = 10) (A) or Olive oil alone (OLO, 5.5 µL/min, n = 9) (B); (iii) Oleate + Bisperoxovanadate (OLE + BPV, oleate-1.3 µmol/min, BPV-0.0025 µmol/kg//min, n = 8) (A) or Olive oil + Bisperoxovanadate (OLO + BPV, OLO-5.5 µL/min, BPV-0.0025 µmol·kg/min, n = 8) (B); or (iv) Bisperoxovanadate alone (BPV, n = 6) (A, B). Data are means ± SEM. *P < .05 vs SAL. &*P* < .05 vs SAL and BPV. &&*P* < .01 vs SAL and BPV. ##*P* < .01 vs BPV. ###*P* < .001 vs BPV.

**Figure 2.**
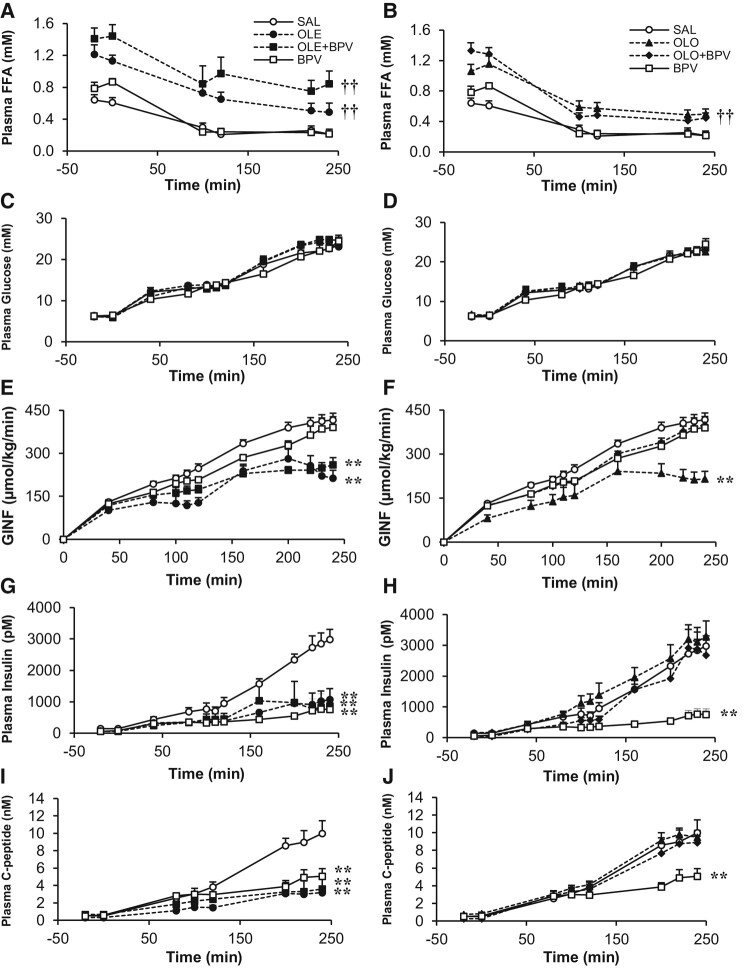
Plasma free fatty acids (FFA; A, B), plasma glucose (C, D), glucose infusion rate (GINF; E, F), plasma insulin (G, H), and plasma C-peptide (I, J) during 2-step hyperglycemic clamps with/without 48 hours oleate (A, C, E, G, I) or olive oil (B, D, F, H, J) intravenous infusion and with/without co-infusion of BPV (A-J) in rats. 12-week-old female Wistar rats were treated with: (i) Saline (SAL, n = 14); (ii) Oleate alone (OLE, 1.3 µmol/min, n = 10) or Olive oil alone (OLO, 5.5 µL/min, n = 9); (iii) Oleate + Bisperoxovanadate (OLE + BPV, OLE:1.3 µmol/min, BPV:0.0025 µmol/kg/min, n = 6) or Olive oil + Bisperoxovanadate (OLO + BPV, OLO-5.5 µL/min, BPV-0.0025 µmol·kg/min, n = 8); or (iv) Bisperoxovanadate alone (BPV, 0.0025 µmol/kg/min, n = 9 for 13 mM and n = 8 for 22 mM). Data are means ± SEM. †† *P* < .01 oleate/olive oil–infused groups vs non-oleate/olive oil–infused groups throughout the hyperglycemic clamp (A, B). **P < .01 OLE and OLE + BPV vs SAL during the second step of the hyperglycemic clamp (E). **P < .01 OLO vs SAL during the second step of the hyperglycemic clamp (F). **P < .01 all vs SAL during the second step of the hyperglycemic clamp (G). **P < .01 BPV vs SAL during the second step of the hyperglycemic clamp (H). **P < .01 all vs SAL during the second step of the hyperglycemic clamp (I). **P < .01 BPV vs SAL during the second step of the hyperglycemic clamp (J).

During the 48 hours infusion period both oleate and olive oil treated rats had higher plasma FFA than rats that were treated with saline or BPV alone (Fig. 1). Following the 48-hour infusions, we evaluated β-cell function in vivo using 2-step (13 and 22 mM) hyperglycemic clamps. As expected, FFA levels decreased during the clamp because of hyperinsulinemia, but remained higher in rats treated with oleate or olive oil (Fig. 2A and 2B). Plasma glucose was similar in all groups in the basal state and during the clamp, as per experimental design (Fig. 2C and 2D). The steady state (last 40 minutes of each clamp step) glucose infusion rate (GINF) necessary to maintain the target glucose level was lower in both oleate and olive oil groups than in the saline-treated group (Fig. 2E and 2F). BPV alone had no effect on GINF. When BPV was added to the oleate infusion, GINF was partially restored during the first step of the hyperglycemic clamp (Fig. 2E), but GINF was not restored during the second step of the clamp. When BPV was used in combination with olive oil, GINF was completely restored to control levels (Fig. 2F). Basal insulin and C-peptide levels were similar in all groups. During the clamp, plasma insulin and C-peptide were lower than saline and similar to oleate in rats treated with BPV alone or BPV in combination with oleate but were similar to control in the groups treated with olive oil or BPV in combination with olive oil (Fig. 2G-2J).

**Figure 3.**
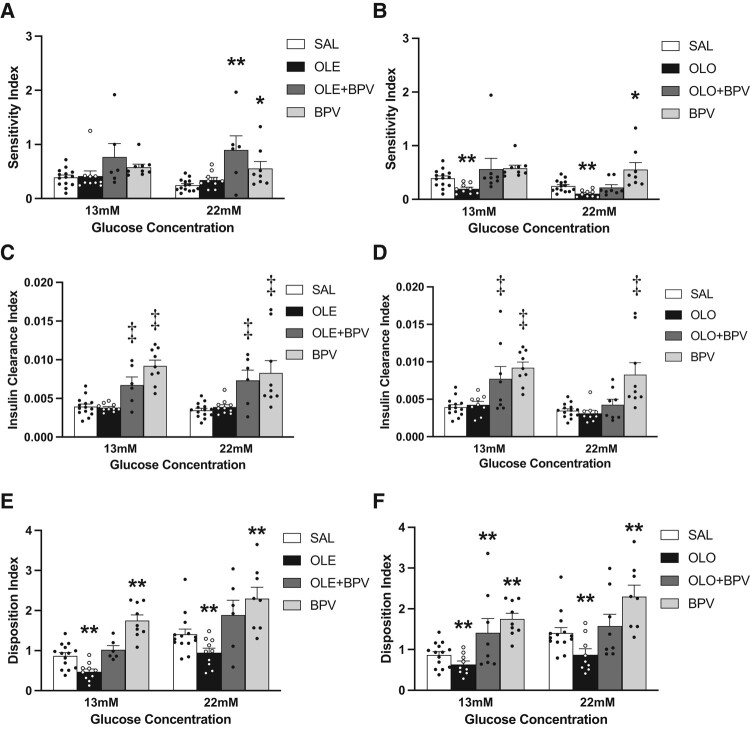
Sensitivity Index (SI = GINF/Insulin, units are μmol/kg/min glucose divided by pM insulin); A, B, Insulin Clearance Index (C-peptide/Insulin, units are nM C-peptide divided by pM insulin); C, D, and Disposition Index (DI = C-peptide multiplied by Sensitivity Index, units are nM C-peptide multiplied by unit of Sensitivity Index); E, F, during 2-step hyperglycemic clamps with/without 48 hours oleate (A, C, E) or olive oil (B, D, F) intravenous infusion and with/without co-infusion of BPV (A-F) in rats. 12-week-old female Wistar rats were treated with: (i) Saline (SAL, n = 14); (ii) Oleate alone (OLE, 1.3 µmol/min, n = 10) or Olive oil alone (OLO, 5.5 µL/min, n = 9); (iii) Oleate + Bisperoxovanadate (OLE + BPV, OLE:1.3 µmol/min, BPV:0.0025 µmol/kg/min, n = 6) or Olive oil + Bisperoxovanadate (OLO + BPV, OLO-5.5 µL/min, BPV-0.0025 µmol·kg/min, n = 8); (iv) Bisperoxovanadate alone (BPV, 0.0025 µmol/kg/min, n = 9 for 13 mM and n = 8 for 22 mM). Data are means ± SEM. *P < .05 vs SAL (A, B). ** *P* < .01 vs SAL (A, B, E, F). ‡ *P* < .05 BPV and OLE + BPV vs SAL or OLE (C, D).

As evidenced by Fig. 3A and 3B and as expected, the sensitivity index, SI, (steady state GINF/plasma insulin) showed a tendency to increase with BPV alone, or in combination with oleate; however, the increases were only significant during the second step of the hyperglycemic clamp. In the group treated with olive oil, the SI was reduced, an effect which was prevented by the addition of BPV. The C-peptide/insulin ratio was calculated as an index of insulin clearance, which is expected to increase with BPV as a result of insulin sensitization. Indeed, in the groups exposed to BPV there was a significant increase in this ratio (Fig. 3C and 3D). The β-cell function calculated as disposition index (DI) was impaired with both oleate and olive oil infusions, but completely restored with the addition of BPV (Fig. 3E and 3F). BPV alone also had an effect to increase the DI throughout the clamp.

#### Ex vivo studies in islets

**Figure 4.**
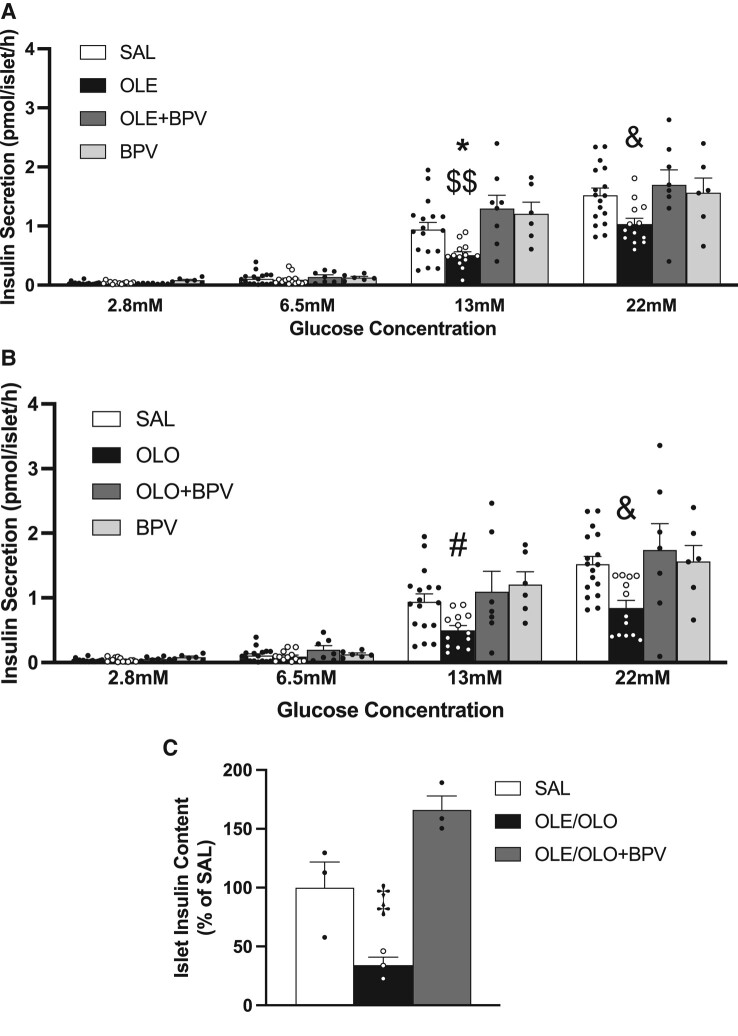
Insulin secretory response to glucose of freshly isolated islets of 12-week-old female Wistar rats intravenously infused for 48 hours with: (i) Saline (SAL, n = 17) (A, B); (ii) Oleate alone (OLE, 1.3 µmol/min, n = 14) (A) or Olive oil alone (OLO, 5.5 µL/min, n = 13) (B); (iii) Oleate + Bisperoxovanadate (OLE + BPV, oleate-1.3 µmol/min, BPV-0.0025 µmol/kg//min, n = 8) (A) or Olive oil + Bisperoxovanadate (OLO + BPV, OLO-5.5 µL/min, BPV-0.0025 µmol·kg/min, n = 7) (B); (iv) Bisperoxovanadate alone (BPV, n = 6) (A, B). Freshly isolated islets of in vivo infused rats were pre-incubated for 1 hour at 37 °C in KRBH supplemented with 2.8 mM glucose. Thereafter, 5 rat islets of approximately the same size were incubated in triplicate in KRBH buffer containing 2.8 mM (non-GSIS), 6.5 mM (basal glucose level in rodents), 13 or 22 mM glucose (as in hyperglycemic clamps) for 2 hours at 37 °C. Insulin content is shown in islets of a random subset of rats intravenously infused as in (A) and (B) (SAL n = 3; OLE/OLO n = 3 [1 OLE, 2 OLO]; OLE/OLO + BPV, n = 3 [2 OLE + BPV, 1 OLO + BPV]) (C). Data are means ± SEM. *P < .05 OLE vs all. $$*P* < .01 OLE vs OLE + BPV and BPV. &*P* < .05 OLE or OLO vs SAL and OLE + BPV or OLO + BPV. #*P* < .05 OLO vs SAL. ‡*P* < .05 OLE/OLO vs SAL and OLE/OLO + BPV.

To directly address the effect of in vivo infusion of BPV on β-cell function independent of peripheral insulin sensitivity and insulin clearance, we evaluated insulin secretion in isolated islets. BPV infusion prevented the GSIS decrease induced by oleate or olive oil infusion at both 13 and 22 mM glucose (Fig. 4A and 4B). No significant effect of BPV alone was observed at any glucose concentration. Insulin content decreased in islets of oleate/olive oil–infused rats but the decrease was prevented by BPV co-infusion (Fig. 4C).

#### In vitro studies in islets

**Figure 5.**
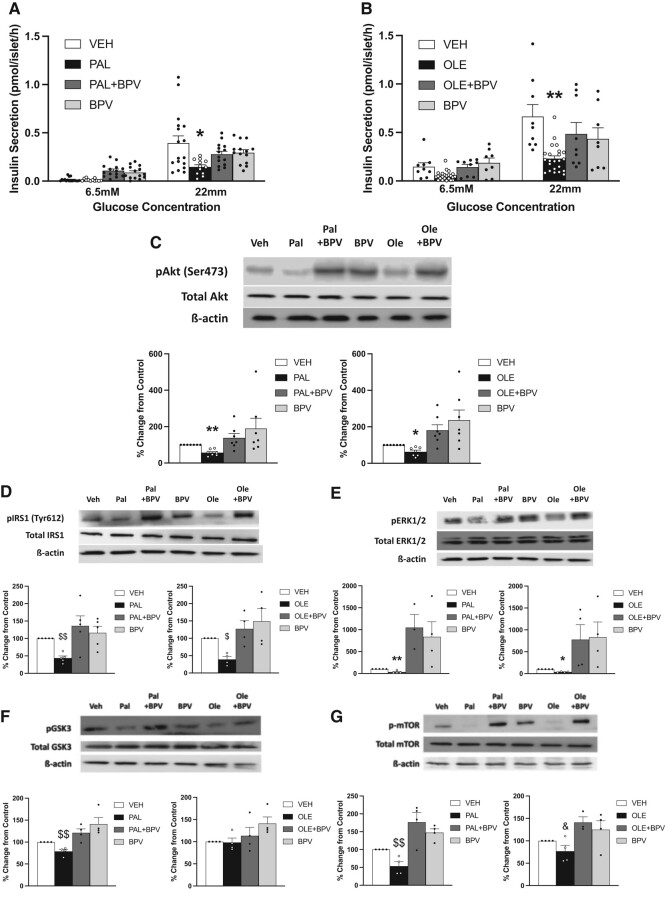
Insulin secretory response to glucose in cultured islets exposed for 48 hours to: (i) bovine serum albumin (BSA, 0.5%; VEH; n = 19); (ii) Palmitate (PAL; 0.4 mM in BSA; n = 14); (iii) Palmitate + Bisperoxovanadate (PAL + BPV; palmitate-0.4 mM in BSA, BPV-4 µM; n = 16); (iv) BPV (n = 15) (A). Insulin secretory response to glucose in cultured islets exposed for 48 hours to: (i) Bovine serum albumin (BSA, 0.5%; VEH; n = 9); (ii) Oleate (OLE; 0.4 mM in BSA; n = 14); (iii) Oleate + Bisperoxovanadate (OLE + BPV; oleate-0.4 mM in BSA, BPV-4 µM; n = 10); (iv) BPV (n = 7) (B). Islets of untreated 12-week-old female Wistar rats were cultured for 48 hours in RPMI 1640 without antioxidants, containing 6.5 mM glucose and 0.4 mM palmitate or oleate in 0.5% BSA or 0.5% BSA alone with or without 4 µM BPV. Thereafter, islets were pre-incubated for 1 hour at 37 °C in KRBH supplemented with 2.8 mM glucose. Subsequently 5 islets of approximately the same size were incubated in triplicate in KRBH buffer containing 6.5 mM or 22 mM glucose for 2 hours for the determination of insulin secretion. Insulin secretion data in (A) and (B) were obtained by 2 different experimenters. Representative blot and quantification of phosphorylated Akt/total Akt expression in cultured islets exposed for 48 hours to VEH, PAL, or OLE with/without BPV (n = 7 for all) after 5-minute 200 nM insulin exposure (C). Representative blots and quantification of other insulin signaling pathway effectors in islets, namely IRS-1, ERK1/2, GSK3β, and mTOR, after 48 hours exposure to the above treatments (n = 4-5). Islets were exposed to 200 nM insulin for 5 minutes before protein quantification (D-G). For (C-G), controls were run for each blot and results of all other groups are expressed as a percentage of control in each blot. Data are means ± SEM. *P < .05 vs all (A, C, E). ** *P* < .01 vs all (B, C, E). $*P* < .05 vs OLE + BPV, BPV (D). $$*P* < .01 vs PAL + BPV, BPV (D, F, G). &*P* < .05 vs OLE + BPV (G).

We performed in vitro studies in cultured islets to investigate whether addition of BPV to palmitate or oleate can prevent fatty acid–induced β-cell dysfunction directly in islets. Similar to our previous studies (28, 32), 0.4 mM palmitate and oleate in 0.5% BSA decreased GSIS (Fig. 5A and 5B). Co-incubation with 4 µM BPV as in previous studies (42) prevented palmitate- and oleate-induced reduction in GSIS, while BPV alone had no significant effect on insulin secretion.

#### Assessment of insulin signaling in pancreatic islet cells

Following 48 hours incubation of rat islets with palmitate or oleate in the presence or absence of BPV, islets were treated with 200 nM insulin for 5 minutes to activate the insulin signaling pathway. Phosphorylation of Akt, IRS-1, ERK1/2, GSK3, and mTOR (marker of activation) was assessed. Both palmitate and oleate significantly reduced insulin-induced Akt activation compared to control. BPV with or without fatty acids tended to increase insulin-induced Akt phosphorylation compared to control (Fig. 5C). Western blots for phosphorylation of other insulin signaling molecules gave consistent results with those of Akt (Fig. 5D-5G).

### Studies in Mice

#### Islet PTEN protein expression and mouse weight

**Figure 6.**
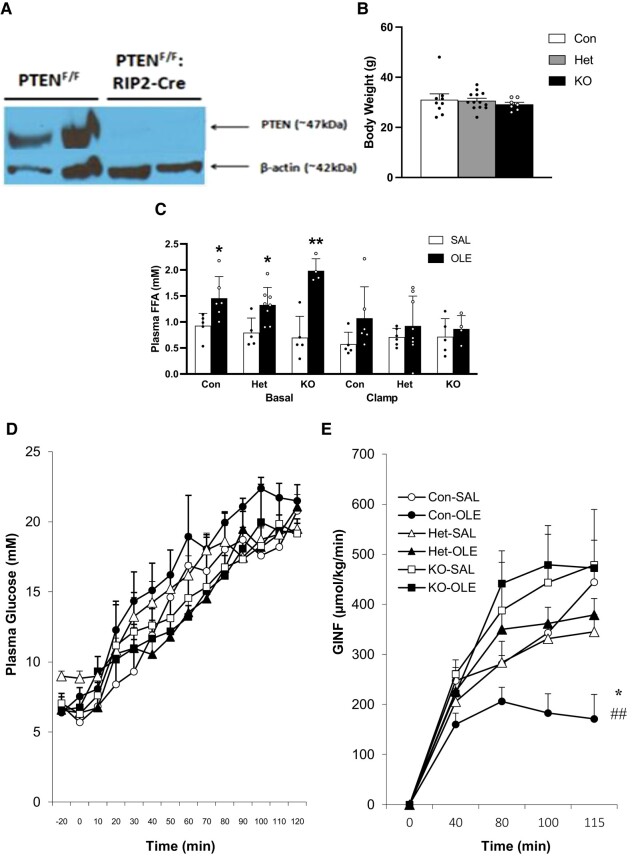
PTEN protein levels in isolated islets of β-cell specific PTEN knockout mice (PTEN^F/F^: RIP2-Cre) and their floxed controls (PTEN^F/F^) (A). Body weights of 12-week-old male β-cell specific homozygous (KO, n = 7) and heterozygous (Het, n = 13) PTEN-knockout mice and littermate controls (mixture of PTEN floxed and wild-type Cre+: Con, n = 9) prior to intravenous infusion (B). Plasma FFA (C) plasma glucose (D), and glucose infusion rate (GINF; E), during a 22 mM hyperglycemic clamp of 12-week-old male β-cell specific homozygous and heterozygous PTEN-knockout mice and littermate controls (mixture of PTEN floxed and wild-type Cre+) infused intravenously for 48 hours with saline (SAL: Con [n = 5], Het [n = 7], KO [n = 5]) or oleate (OLE: 0.4 µmol/min; Con [n = 6], Het [n = 8], KO [n = 5]). Data are means ± SEM. * *P* < .05 vs respective SAL; ** *P* < .01 vs respective SAL (C). * *P* < .05 vs Con-SAL; ## *P* < .01 vs KO-OLE (E).

PTEN protein was expressed in control islets and undetectable in islets of β-cell specific homozygous PTEN-knockout mice (Fig. 6A). Unexpectedly, there were no significant differences in weight between control, β-cell specific heterozygous PTEN-knockout mice, or β-cell specific homozygous PTEN-knockout mice (Fig. 6B).

#### In vivo hyperglycemic clamps

Following 48-hour oleate infusion and prior to the hyperglycemic clamps, plasma FFA levels were higher in oleate-infused groups than in their saline-infused counterparts, and they decreased during the clamp as expected (Fig. 6C).

**Figure 7.**
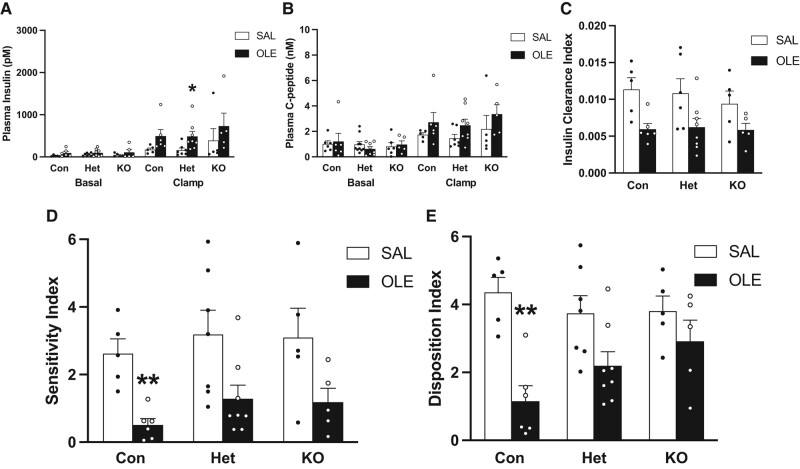
Plasma Insulin (A), Plasma C-peptide (B), Insulin Clearance Index (C-peptide/Insulin, units are nM C-peptide divided by pM insulin) (C), Sensitivity Index (SI = GINF/Insulin, units are μmol/kg/min glucose divided by pM insulin) (D), and Disposition Index (DI = C-peptide multiplied by Sensitivity Index, units are nM C-peptide multiplied by unit of Sensitivity Index) (E) during a 22 mM hyperglycemic clamp of 12-week-old male β-cell specific homozygous and heterozygous PTEN-knockout mice (KO and Het, respectively) and littermate controls (mixture of PTEN floxed and wild-type Cre+, Con) infused i.v. for 48 hours with saline (SAL: Con [n = 5], Het [n = 7], KO [n = 5]) or oleate (OLE: 0.4 µmol/min; Con [n = 6], Het [n = 8], KO [n = 5]). * *P* < .05 vs respective SAL. ** *P* < .01 vs respective SAL.

Glucose levels were raised to a steady state level of ∼22 mM during the last 20 minutes of the clamp in all groups (Fig. 6D). The steady state glucose infusion rate (GINF) was decreased in oleate-treated control mice (PTEN floxed and wild-type Cre+) compared to saline-treated controls. This decrease was prevented in oleate-treated β-cell specific homozygous and heterozygous PTEN-knockout mice (Fig. 6E). Basal insulin (Fig. 7A) and C-peptide (Fig. 7B) levels did not differ between groups. During the clamp both insulin and C-peptide levels tended to be higher in each oleate-infused group compared to its respective saline-infused group (Fig. 7A and 7B).

The index of insulin clearance tended to be decreased in each of the oleate-infused groups compared to its respective saline-infused group, but significance was not reached in any group (Fig. 7C). The sensitivity index for each oleate-infused group tended to be decreased compared to its respective saline-infused group, but significance was not reached in homozygous or heterozygous knockout mice (Fig. 7D). In contrast, the sensitivity index in the control mice infused with oleate was significantly decreased compared to their saline-infused counterparts. The DI was significantly decreased in oleate-treated control mice compared to saline-treated controls. This decrease was partially prevented in oleate-treated β-cell specific homozygous and heterozygous PTEN-knockout mice (Fig. 7E).

#### Ex vivo studies in islets and % β-cell/total pancreatic area

**Figure 8.**
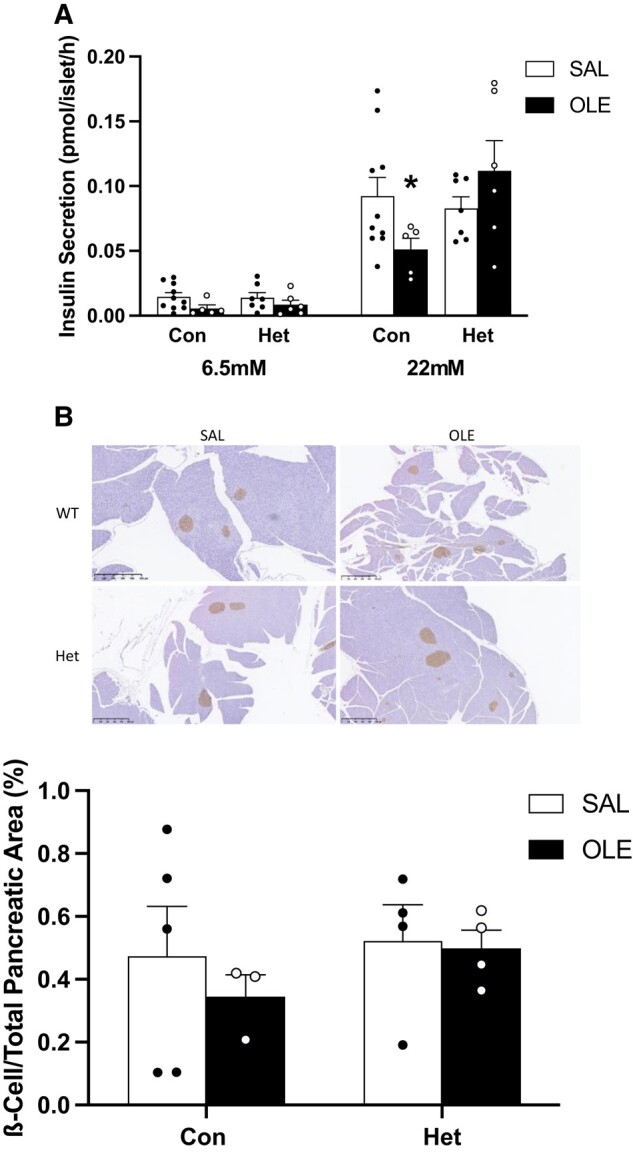
Insulin secretion (A) in islets isolated from 12-week-old male β-cell specific heterozygous PTEN-knockout mice and littermate controls (mixture of PTEN floxed and wild-type Cre+) infused intravenously for 48 hours with saline (SAL: Con [n = 10], Het [n = 7]) or oleate (OLE: 0.4 µmol/min; Con [n = 5], Het [n = 6]). Freshly isolated islets of in vivo infused mice were pre-incubated for 1 hour at 37 °C in KRBH supplemented with 2.8 mM glucose. Thereafter, 10 mouse islets of approximately the same size were incubated in duplicate at 6.5 and 22 mM glucose for 2 hours at 37 °C. Representative images and quantification of % β-cell/total pancreatic area (B) in heterozygous 12 week old male PTEN-knockout mice and littermate controls (mixture of PTEN floxed and wild-type Cre+) infused intravenously for 48 hours with saline (SAL: Con [n = 5], Het [n = 4]) or oleate (OLE: 0.4 µmol/min; Con [n = 3], Het [n = 4]). Data are means ± SEM. * *P* < .05 vs respective SAL.

To directly address the effect of oleate infusion in vivo on β-cell function independent of peripheral insulin sensitivity and insulin clearance, we performed ex vivo studies in isolated islets of control and β-cell specific heterozygous mice. β-cell specific homozygous PTEN-knockout mice were not used for ex vivo experiments, as in vivo studies did not show any significant differences in β-cell function between the homozygous and heterozygous knockout mice. Islets of control mice (PTEN-floxed and wild-type Cre-positive mice were used since there was no difference in their insulin secretion) infused with oleate had decreased levels of GSIS compared to saline. This decrease was completely prevented in oleate-infused β-cell specific heterozygous PTEN-knockout mice (Fig. 8A). Although % β-cell/total pancreatic area (index of β-cell mass) appeared to be lower in oleate-treated control mice than in saline-treated control and both saline- and oleate-treated heterozygous PTEN-knockout mice, none of these differences was significant (*P* > .2) (Fig. 8B).

## Discussion

In vitro studies have shown that fatty acids impair β-cell insulin signaling (21-24) and insulin gene transcription (24) while studies by some of our group have demonstrated that β-cell specific deletion of a negative regulator of insulin signaling (ie, PTEN) protects against high fat diet–induced diabetes (25). However, whether upregulation of β-cell insulin signaling protects against β-cell dysfunction specifically induced by circulating fatty acids has not been investigated in an in vivo model.

In this study, we show that the insulin mimetic tyrosine phosphatase inhibitor bisperoxovanadate (BPV), which upregulated insulin signaling, prevented the impairment in β-cell function induced by prolonged FFA exposure in vivo in rats and ex vivo and in vitro in rat islets. We also show that β-cell specific PTEN deletion protects against β-cell dysfunction induced selectively by prolonged FFA exposure in vivo and ex vivo in mice and mouse islets, respectively. Together, these data support a causal role of β-cell insulin resistance in β-cell dysfunction specifically induced by fat.

In our in vivo clamp studies in rats, both insulin and C-peptide responses to glucose were lower in rats treated with oleate, but not olive oil as in our previous study (28). This is because with olive oil, the β-cell attempted to compensate for insulin resistance. The insulin resistance induced by olive oil may be due to the amount (16%) of saturated fat contained in olive oil, as opposed to oleate, which is a monounsaturated fatty acid, and also to the effect of the triglycerides of olive oil on insulin resistance (28).

As expected, insulin sensitivity was increased by BPV and the olive oil induced insulin resistance was prevented. An even greater effect of BPV was seen on increasing insulin clearance. Since insulin clearance is mainly a liver function and is dependent on insulin receptor tyrosine phosphorylation, these results suggest that liver is a primary target tissue for BPV. When disposition index (DI) was used to assess β-cell function, there was a decrease with both oleate and olive oil infusions. This effect was completely prevented by addition of BPV to both fat infusions. An interesting finding was the effect of BPV alone, which increased β-cell function in vivo, as measured by the DI but did not affect β-cell function ex vivo or in vitro. β-cell function in vivo must be considered not only in relationship with insulin resistance, but also with insulin clearance. Since BPV alone increased C-peptide/insulin ratio (index of insulin clearance), β-cells presumably had to secrete more to compensate for insulin disappearance. In the oleate-treated rats despite normalizing the DI, co-infusion of BPV did not normalize GINF. This is likely because at 22 mM insulin clearance increased in the group treated with oleate plus BPV more than the β-cell could compensate for. Insulin clearance did not increase at 22 mM in the olive oil plus BPV group, likely because the effect of BPV to increase clearance was counteracted by the effect of attempted compensation for olive oil induced insulin resistance, which would tend to decrease insulin clearance. Since insulin clearance did not change in the olive oil plus BPV group, both GINF and DI were normalized.

To assess β-cell function independent of systemic factors influencing the β-cell response to glucose in vivo (ie, the prevailing insulin sensitivity and insulin clearance increased by BPV), we examined GSIS ex vivo in isolated islets. Furthermore, to eliminate any indirect effect of in vivo treatments in the 48 hours preceding islet isolation on GSIS, we exposed islets directly to fatty acids and BPV in vitro. We found that 48-hour infusion of oleate or olive oil impaired GSIS ex vivo in isolated islets, consistent with our in vivo findings and with our previous study (28). 48 hours infusion of BPV protected the islets from the fat-mediated impairment in GSIS. Direct exposure of islets for 48 hours to palmitate or oleate decreased the insulin response at high glucose and in vitro exposure to BPV restored GSIS, similar to our ex vivo model. Fatty acid exposure also reduced insulin-induced phosphorylation of Akt and other insulin signaling molecules, which was counteracted by BPV. These results are in line with previous studies that showed impaired Akt activation by FFA (21, 23). The effect of BPV to increase Akt activation implicates upregulation of the insulin signaling pathway in β-cells as a mechanism through which β-cell dysfunction induced by fat may be prevented. Consistent with this notion, we previously demonstrated that exposure to oleate results in IRS-1 serine-307 phosphorylation in islets (28). We have also previously shown and confirmed by our current results that fat-induced GSIS impairment is primarily due to reduced islet insulin content (31, 36), which is in keeping with decreased proinsulin biosynthesis induced by reduced insulin gene transcription by FOXO1 (11) and reduced proinsulin translation due to decreased mTOR (44) in the presence of impaired insulin signaling.

Previously, BPV has been used in vitro in islets of diabetic GK rats, where it was found to increase both basal as well as glucose-stimulated insulin secretion. However, it only increased insulin secretion at basal or low stimulatory glucose levels in islets of control Wistar rats, an effect prevented by the PI3K inhibitor wortmannin (42, 45). Other authors showed that 72-hour treatment with vanadate improved GSIS (46). No study to our knowledge has used vanadium compounds in combination with fatty acids.

Since BPV is a nonspecific tyrosine phosphatase inhibitor and has systemic effects when used in vivo, we used another model of upregulation of β-cell insulin signaling, that is, the β-cell specific PTEN (negative regulator of insulin signaling) knockout mouse for further in vivo studies. In contrast to previous findings (25, 30), our homozygous knockout mice did not show growth retardation. The discrepant findings between our study and previous studies by some of us (25, 47) may be explained by different expression of PTEN in the hypothalamus and/or by the difference in the genetic mix of C57BL/6J vs 129J in our colony that was not deliberately maintained on a C57BL/6-129J mixed background, unlike that of the previous studies (25, 47).

β-cell specific PTEN-knockout mice were reported to have increased insulin signaling in β-cells and were protected against glucose intolerance induced by high fat diet and diabetes induced by leptin receptor deficiency or streptozotocin (25, 30, 47). The previous studies in high fat diet–fed mice support a role for β-cell insulin resistance in fat-induced metabolic dysfunction, but they did not investigate the role of β-cell insulin resistance in the β-cell dysfunction induced selectively by elevated circulating levels of fat.

In order to better understand the effect of oleate on β-cell function, and in the context of the ambient insulin sensitivity, insulin and C-peptide levels were assessed during hyperglycemic clamps and used to calculate the sensitivity index SI and the disposition index DI. Both insulin and C-peptide levels tended to increase in oleate-infused mice compared to their saline-infused counterparts, which is consistent with the previously demonstrated effect of oleate to induce insulin resistance in mice (similar to olive oil in rats) (28, 36) and thereby cause β-cells to increase the level of insulin secretion in an attempt to compensate. In line with this, the insulin sensitivity index was decreased in control mice infused with oleate. This was partially prevented in both heterozygous and homozygous β-cell specific PTEN-knockout mice. This is not entirely in agreement with previous studies by some of our group that showed that β-cell specific PTEN-knockout mice have enhanced insulin sensitivity and are completely protected against insulin resistance induced by high fat diet due to neuronal PTEN deficiency (25, 47). In addition to different degrees of neuronal PTEN deficiency, the discordant results may be due to (i) the greater C57BL/6J genetic mixture in the background of our colony, which would be consistent with the susceptibility of C57BL/6J mice to insulin resistance; (ii) to the fact that our mice did not show growth retardation; or (iii) to differences in age as mice in Nguyen's study were 8 to 10 weeks old (47), whereas mice in ours were 11 to 13 weeks old. Interestingly, the mice studied by Stiles et al, who also found no significant changes in insulin sensitivity in β-cell specific PTEN-knockout mice (30), had similar age to ours and were also not deliberately maintained on a mixed background.

DI was decreased in oleate-infused control mice compared to saline-infused controls and this decrease was partially prevented in both heterozygous and homozygous β-cell specific PTEN-knockout mice. We have previously found that heterozygous PTEN knockout mice have reduced islet PTEN expression (unpublished) and a metabolic phenotype intermediate between homozygous PTEN knockout mice and controls (47). Protection against fat-induced β-cell dysfunction is in line with previous findings obtained with high fat diet (25). In the present study, however, according to the DI, mice were only partially protected against fat-induced β-cell dysfunction.

In order to determine the role of β-cell insulin signaling on insulin secretion specifically, independent of effects of insulin sensitivity and insulin clearance, we performed ex vivo insulin secretion studies. Insulin secretion was decreased in islets of control mice infused with oleate and this decrease was prevented in islets of β-cell specific heterozygous PTEN-knockout mice. In the ex vivo islet studies, prevention of β-cell dysfunction was complete even in heterozygous mice, presumably indicating that part of the DI decrease in vivo was due to β-cell rest because insulin levels were already elevated by the oleate-induced decrease in insulin clearance. The in vivo and ex vivo data in this mouse model, taken together, support the hypothesis that upregulation of insulin signaling in β-cells prevents fat-induced β-cell dysfunction. We have previously shown that homozygous β-cell specific PTEN-knockout mice have greater % β-cell/total pancreatic area (ie, β-cell mass) than controls when fed chow or high fat diet; however, in response to high fat diet, their % β-cell/total pancreatic area does not further increase, unlike that of control mice. In contrast, β-cell function evaluated by islet perifusion, which is similar to controls in chow-fed mice, is markedly greater in high fat diet–fed PTEN knockout mice that are protected from the high fat diet–induced decrease in function. Thus, the greater insulin secretion in vivo in high fat diet–fed PTEN knockout mice is attributable to greater β-cell function (25) in addition to their still increased % β-cell/total pancreatic area (ie, β-cell mass). A 48-hour oleate infusion does not result in changes in % β-cell/total pancreatic area or β-cell mass in controls (36), presumably because of relatively brief FFA elevation, and in the present study, did not significantly change % β-cell/total pancreatic area in either controls or heterozygous PTEN mice, despite these mice appearing to have greater β-cell/total pancreatic area than controls when treated with oleate. In our GSIS experiments, the same number of islets of approximately the same size were used in controls and heterozygous PTEN knockout mice or BPV-treated rats or rat islets, and the results indicate that β-cell upregulation of insulin signaling did protect against oleate-induced β-cell secretory dysfunction in all models. Also, in rats, 48-hour BPV administration is unlikely to change β-cell mass. Thus, the β-cell protection that upregulation of insulin signaling provided in both rats and mice is mainly attributable to an increase in β-cell secretory function, although we cannot exclude a contribution of % β-cell/total pancreatic area (ie, β-cell mass) in PTEN knockout mice. In addition, studies from other groups have shown that functional impairment is sufficient to explain increased susceptibility to β-cell failure by high fat diet in inducible model of β-cell insulin receptor deficiency (48).

Although previous in vitro studies (14-19) and a recent in vivo study (49) have yielded conflicting results, the majority of studies performed in vivo implicate an essential role of β-cell insulin signaling in β-cell function as βIRKO mice (20, 50) as well as mice expressing a mutant Akt in β-cells (51) have impaired insulin secretion. In humans, exogenous insulin administration, although it decreases concomitant endogenous insulin secretion by a neural reflex (52), has been shown to potentiate subsequent GSIS (53-55).

In summary, we show that both pharmacologic and genetic upregulation of insulin signaling in rodents protects against fat-induced β-cell dysfunction in vitro, ex vivo, and in vivo. This supports the hypothesis that β-cell insulin resistance plays a causal role in fat-induced β-cell dysfunction.

## Data Availability

All data are available from the corresponding author on reasonable request.

## Abbreviations

Akt: protein kinase B
BPV: bisperoxovanadate
BSA: bovine serum albumin
DI: disposition index
FFA: free fatty acids
GINF: glucose infusion rate
GSIS: glucose-stimulated insulin secretion
IRS: insulin receptor substrate
KRBH: Krebs-Ringer bicarbonate HEPES
OLE: oleate
OLO: olive oil
PI3K: phosphoinositide 3-kinase
PIP3: phosphatidylinositol-34,5-trisphosphate
PTEN: phosphatase and tensin homolog
SAL: saline control
SEM: standard error of the mean
SI: sensitivity index
